# *Klebsiella pneumoniae* Complex Harboring *mcr-1*, *mcr-7,* and *mcr-8* Isolates from Slaughtered Pigs in Thailand

**DOI:** 10.3390/microorganisms9122436

**Published:** 2021-11-25

**Authors:** Nattamol Phetburom, Parichart Boueroy, Peechanika Chopjitt, Rujirat Hatrongjit, Yukihiro Akeda, Shigeyuki Hamada, Suphachai Nuanualsuwan, Anusak Kerdsin

**Affiliations:** 1 Faculty of Public Health, Kasetsart University, Chalermphrakiat Sakon Nakhon Province Campus, Sakon Nakhon 47000, Thailand; nattamol.phet@ku.th (N.P.); peechanika.c@ku.th (P.C.); anusak.ke@ku.th (A.K.); 2 Faculty of Science and Engineering, Kasetsart University, Chalermphrakiat Sakon Nakhon Province Campus, Sakon Nakhon 47000, Thailand; rujirat.ha@ku.th; 3 Japan-Thailand Research Collaboration Center for Infectious Diseases, Research Institute for Microbial Diseases, Osaka University, Suita, Osaka 565-0871, Japan; akeda@biken.osaka-u.ac.jp (Y.A.); hamadas@biken.osaka-u.ac.jp (S.H.); 4 Department of Infection Control and Prevention, Graduate School of Medicine, Osaka University, Suita, Osaka 565-0871, Japan; 5 Division of Infection Control and Prevention, Osaka University Hospital, Osaka University, Suita, Osaka 565-0871, Japan; 6 Department of Veterinary Public Health, Faculty of Veterinary Science, Chulalongkorn University, Bangkok 10330, Thailand; suphachai.n@chula.ac.th; 7 Food Risk Hub, Research Unit of Chulalongkorn University, Bangkok 10330, Thailand

**Keywords:** *Klebsiella pneumoniae*, *mcr*, colistin, pigs, Thailand

## Abstract

Dissemination of the mobile colistin resistance gene *mcr* in *Enterobacterales* among humans, animals, and the environment is a public health issue. We characterized *mcr* genes in the *Klebsiella* *pneumoniae* complex (KpnC) isolated from slaughtered pigs in Thailand. The 280 KpnCs consisted of *K. pneumoniae* (85%), *Klebsiella quasipneumoniae* (8.21%), and *Klebsiella variicola* (6.79%). *mcr* genes were detected in 6.79% (19/280) of KpnC isolates, consisting of *mcr-8* (*n* = 9; 3.21%), *mcr-7* (*n* = 7; 2.50%), *mcr-7* + *mcr-8* (*n* = 2; 0.71%), and *mcr-1* + *mcr-7* (*n* = 1; 0.36%). *K. pneumoniae* predominantly carried the *mcr-7* and *mcr-8* genes, while *K. variicola* and *K. quasipneumoniae* harbored *mcr-7* and *mcr-8,* respectively. Six of the nineteen *mcr*-harboring KpnC isolates exhibited colistin resistance, and five had *mcr-1* or *mcr-8* transferable to an *Escherichia coli* recipient. Antimicrobial susceptibility analysis revealed that all *mcr*-carrying KpnC isolates were susceptible to carbapenems, cefotaxime, cefepime, amoxicillin/clavulanic acid, piperacillin/tazobactam, amikacin, and fosfomycin, and had high resistance to azithromycin. Multilocus sequence analysis demonstrated that the *mcr*-harboring KpnC isolates were genetically diverse. A ‘One-Health’ approach is useful to combat antimicrobial-resistant bacteria through coordinating the human, animal, and environmental sectors. Hence, continuous monitoring and surveillance of *mcr-*carrying KpnCs throughout the pork supply chain is crucial for ensuring public health.

## 1. Introduction

The *Klebsiella pneumoniae* complex (KpnC) comprises five closely related species: *K. pneumoniae*, *K. quasipneumoniae* subsp. *quasipneumoniae*, *K. quasipneumoniae* subsp. *similipneumoniae*, *K. variicola* subsp. *variicola, K. variicola* subsp. *tropica, K. africana*, and *K. quasivariicola* [1,2,3,4]. KpnC is one of the major sources of multidrug resistance (MDR), especially carbapenem resistance, which affects humans, with hospital infections being associated with high morbidity and mortality [5]. Due to limited treatment options for carbapenem-resistant KpnC (CRKpnC), colistin has become a ‘last-in-line’ therapeutic drug.

The increased use of colistin has led to the emergence of resistance to colistin in *K. pneumoniae* worldwide, which has become a serious public health problem associated with high morbidity and mortality rates among humans and animals [6,7,8]. Colistin resistance is generally thought to be a mutation of lipopolysaccharide modification genes such as *mgrB*, *phoP/phoQ, pmrA/pmrB, crrA/crrB, qseB/qseC, yciM,* and *lpxM* in chromosomes [9,10]. The mobile colistin resistance gene (*mcr-1*) was first discovered in *Escherichia coli* from pigs in China [11]. To date, 10 *mcr* variants (*mcr-1* to *mcr-10*) have been reported [12,13]. Among the 10 *mcr* variants, *mcr-1* is widely distributed in many bacterial species, such as *E*. *coli*, *K*. *pneumoniae*, *Enterobacter cloacae*, *Salmonella enterica*, *Shigella* spp., *Citrobacter freundii*, *Kluyvera ascobarta*, *Raulotella ornitholytica*, *Proteus mirabilis*, *Acinetobacter lwoffi*, *Pseudomonas* spp., and *Aeromonas* spp. [14]. In contrast, *mcr-2* through *mcr-10* have been reported in limited bacterial species, specifically *E. coli* (*mcr-2* through *mcr-4* and *mcr-9*), *Salmonella enterica* (*mcr-4*, *mcr-5,* and *mcr-9*), *K*. *pneumoniae (mcr*-*7*, *mcr*-*8*, and *mcr*-*9)*, *Enterobacter hormaechei* (*mcr*-*9*), *Enterobacter roggenkampii* (*mcr-10*), *Moraxella* spp. *(mcr*-*1* and *mcr*-*2)*, and *Acinetobacter* spp. (*mcr-1* and *mcr-4*) [13,15,16,17].

The *mcr* genes have been reported worldwide in *Enterobacterales* (including *E. coli*, *Salmonella*, and *K. pneumoniae*) from various sources, especially the environment and in animals [18]. *E. coli* is the most prevalent species among the *mcr*-harboring isolates reported so far, accounting for approximately 91% of the total *mcr*-carrying isolates, followed by *Salmonella enterica* (~7%) and *K. pneumoniae* (~2%) [19]. The *mcr* gene has been detected in 47 different countries across six continents, including developed and developing countries. It was identified from human sources in 44 countries; livestock in 21 countries; meat and food products in 13 countries; and from other sources, including pets, exotic or wild animals, and the environment, in 11 countries [19]. 

Livestock, especially pigs, are considered a reservoir of colistin-resistant organisms because colistin is approved in pig production in several countries with different purposes, including for the control of *Enterobacterales* infections therapeutically, prophylactically, and even for growth promotion [20]. Pigs maintain the microorganism on the farm, contaminating their environment and cross-contaminating carcasses during slaughter, which poses a potential risk to humans through co-circulation in human and swine populations, while transmission or spillover into humans can occur via tasting or eating undercooked products or preparing meals at home [21]. The prevalence of *mcr-1-*harboring bacteria has been found to range from 0.35 to 36.00% in pigs; however, some studies reported very high positivity rates in pigs ranging from 13.20 to 98.00% [22]. Recent studies in Thailand showed the presence of several *mcr* genes (*mcr-1*, *mcr-3*, *mcr-6*, *mcr-7*, *mcr-8*, and *mcr-9*, together with co-occurrences of *mcr-1 + mcr-3*, *mcr-1 + mcr-9*, and *mcr-3 + mcr-6 + mcr-7*) in *E. coli* isolated from slaughtered pigs, as well as *mcr-1* or *mcr-3* being detected in either *E. coli* or *K. pneumoniae* isolated from humans [23,24]. Two mechanisms of *mcr-1* transmission have been recognized: horizontal gene transfer (HGT) and clonal transmission [24]. The transferability of the *mcr*-carrying plasmid from *E. coli*, *K. pneumoniae*, *Klebsiella aerogenes*, *Salmonella enterica*, *Enterobacter cloacae*, or *Cronobacter sakazakii* isolates of either animal or human origin to humans by HGT was demonstrated by in vitro conjugation or transformation experiments, showing the successful transfer of an *mcr*-*1* plasmid from animal or human origin into common human pathogenic *Enterobacteriaceae* and *Pseudomonas aeruginosa* [11,19,25]. Thus, *mcr-1* in uncommon *Enterobacteriaceae* strains can be transferred into common *Enterobacteriaceae*, being further disseminated and circulated among environmental or human *Enterobacteriaceae* species. Clonal transmission has indicated that *mcr-1*-harboring isolates from food animals could be transferred to humans mainly through the food chain or direct contact, showing that the same *E. coli* STs carrying *mcr-1* were detected in food animals (on farms and in slaughterhouses), animal products (in markets and supermarkets), and humans (healthy populations and patients). Core-genome, single-nucleotide polymorphism (SNP)-based phylogenetic analysis, and XbaI pulsed-field gel electrophoresis (PFGE) analysis further supported the commonality of *mcr-1*-harboring isolates among disparate samples [22].

We characterized KpnC harboring *mcr* isolated from slaughtered pigs in Thailand by showing the predominant *mcr-7* and *mcr-8* in these isolates, antimicrobial susceptibility patterns, the genetic diversity of KpnC harboring *mcr* isolates, and the horizontal transferability of these *mcr* genes. The results provide evidence that slaughtered pigs are a reservoir of *mcr*-7 and *mcr-8* for subsequent dissemination. This research will strengthen the evidence-based knowledge of *mcr*-harboring KpnC in slaughtered pigs and will contribute to strategic planning for the control of overuse or misuse of antimicrobial drugs on farms and the prevention of this pathogen contaminating farmed livestock, as well as assisting surveillance on this organism.

## 2. Materials and Methods

### 2.1. Bacterial Strains and Identification

In total, 280 KpnC isolates were collected and isolated from the carcasses of slaughtered pigs across 10 provinces in Thailand during 2014 and 2015. Four slaughterhouses were randomly selected from each province, with 50 swab samples randomly collected from each slaughterhouse, resulting in a sample size of 2000 swab samples. One side of the carcass was swabbed for a total area of 400 cm^2^ using a single swab. The swab samples were immediately stored on ice in zip-lock bags throughout transportation to the microbiological laboratory. Isolation and identification of *K. pneumoniae* were carried out using a 10-fold serial dilution of swab samples achieved using buffered peptone water (BPW). Each diluted BPW was spread onto MacConkey agar and incubated at 37 °C for 24 h. The presumptive *Klebsiella* species for up to five colonies were confirmed using conventional biochemical tests described elsewhere [26]. All *Klebsiella* isolates were stored at −80 °C in a laboratory freezer until used in this study.

All isolates from the freezer were cultured on MacConkey agar, and their DNA was prepared using ZymoBIOMICS™ DNA Miniprep kits (Zymo Research Corp., Irvine, CA, USA) following the manufacturer’s instructions. Multiplex polymerase chain reaction (PCR) identification of species in KpnC (*K. pneumoniae*, *K. variicola*, and *K. quasipneumoniae*) was carried out as described previously [27], with certain modifications involving replacing the former PCR primers for *K. pneumoniae* with primers for the *K. pneumoniae waaQ* gene, as described previously [28]. The PCR program involved initial denaturation at 95 °C for 3 min, followed by 35 cycles of denaturation at 95 °C for 30 s, and annealing and extension at 65 °C for 1 min. PCR of the *Kp50233* gene was used for confirmation of the *K. pneumoniae*-positive samples [29]. 

### 2.2. Detection of Antimicrobial Resistance Genes

The *mcr-1* to *mcr-9* gene variants were identified using PCR as described previously [23]. The carbapenemase genes (*bla*_IMP_, *bla*_KPC_, *bla*_NDM_, and *bla*_OXA-48-like_), β-lactamase genes (*bla*_CTX-M_, *bla*_TEM_, and *bla*_SHV_), and plasmid-mediated quinolone resistance (PMQR) genes were identified using multiplex PCR [30,31,32]. The PCR products of the *mcr* genes were subjected to Sanger DNA sequencing for confirmation.

### 2.3. Antimicrobial Susceptibility Testing

All techniques were performed and interpreted according to the 2021 Clinical and Laboratory Standards Institute (CLSI) guidelines [33]. KpnC isolates positive for *mcr* were further investigated for antimicrobial susceptibility using the disk diffusion method to provide evidence-based guidance for further optimizing effective antimicrobial treatment options and surveillance for the emergence of antibiotic drug resistance. Antimicrobial disks used in the assay were loaded with ampicillin, gentamicin, amikacin, amoxicillin/clavulanic acid, piperacillin/tazobactam, cefepime, cefotaxime, ceftazidime, ertapenem, imipenem, meropenem, ciprofloxacin, levofloxacin, chloramphenicol, tetracycline, fosfomycin, nitrofurantoin, azithromycin, or trimethoprim. *E. coli* ATCC 25922 was used as a control. The minimal inhibitory concentration (MIC) of colistin was determined using the broth microdilution method. 

### 2.4. Multilocus Sequence Typing (MLST)

To explore the genetic diversity of KpnC isolates from pigs in this study, MLST was performed according to the Pasteur scheme of Institut Pasteur (https://bigsdb.pasteur.fr/klebsiella/klebsiella.html accessed on 24 September 2021). The PCR products of the seven housekeeping genes were purified using an E-Z 96 Cycle Pure Kit (Omega, GA, USA) following the manufacturer’s instructions. Sanger DNA sequencing of the purified PCR products was performed by Apical Scientific Sdn Bhd, Selangor, Malaysia. MLST alleles and the resulting sequence types (STs) were identified using the Institute Pasteur MLST database (https://bigsdb.pasteur.fr/klebsiella/klebsiella.html accessed on 24 September 2021). Novel alleles or allelic profiles were submitted to the curator of the database to assign an allele or ST number. The PHYLOViZ 2.0 software [34] was used to analyze STs and their clonal complexes (CCs) [35]. 

### 2.5. Conjugation Assays

Conjugation assays were carried out using all isolates of *mcr-*harboring KpnC strains (donor) with streptomycin-resistant *E. coli* UB1637 (recipient), as described elsewhere [36]. All *E. coli* transconjugants were then selected on the basis of growth on MacConkey agar containing 1 μg/mL of colistin and 3200 μg/mL of streptomycin. Transconjugants were confirmed as *E. coli* using PCR [37] and for the presence of antimicrobial-resistance genes consisting of mobile colistin resistance (*mcr-1* to *mcr-9*) genes, β-lactamase genes (*bla*_TEM_, *bla*_SHV_, and *bla*_CTX-M_), and plasmid-mediated quinolone resistance (PMQR) genes using PCR, as described above. Colistin MIC values were determined as described above. Details concerning the donor strains used in conjugation assays are shown in Table 1.

## 3. Results

### 3.1. Identification of Klebsiella Species and Antimicrobial Resistance Genes in KpnC Isolates

The 280 KpnC isolates from slaughtered pigs were identified as 238 *K. pneumoniae* (85%), 19 *K. variicola* (6.79%), and 23 *K. quasipneumoniae* (8.21%). Nineteen isolates (6.79%) carried *mcr* genes, with *K. pneumoniae* (16/19) the most predominant among these. Among the *mcr* genes, *mcr-8* (8/280) was mainly found in *K. pneumoniae*, followed by *K. quasipneumoniae* (1/280), while *mcr-7* (7/280) was found in *K. pneumoniae* (6/280) and *K. variicola* (1/280). Co-existing *mcr* genes were found, namely, *mcr-7* and *mcr-8* in *K. pneumoniae* (2/280) and *mcr-1* and *mcr-7* in *K. quasipneumoniae* (1/280). In the current study, no carbapenemase genes were detected in any isolate. The KpnC isolates harbored other β-lactamase genes in 248 of the 280 isolates examined. Among *K. pneumoniae* (*n* = 238), we detected *bla*_SHV_ (223/238; 93.69%), *bla*_TEM_ (40/238; 16.80%), and *bla*_CTX-M_ (7/238; 2.94%) (Table 1). The *bla*_CTX-M_ gene was detected in 13 isolates, approximately half of which belonged to *K. variicola* (6/13; Table 1). PMQR genes *oqxAB* (168/238; 70.59%), *qnrS* (4/238; 1.68%), and *oqxAB+ qnrS* (36/238; 15.13%) were detected in *K. pneumoniae* isolates.

### 3.2. Antimicrobial Susceptibility Assays

**Figure 1:**
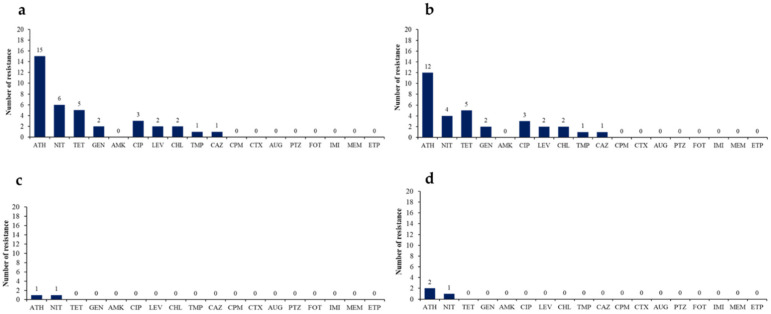
Antimicrobial resistance of KpnC isolates from slaughtered pigs: (**a**) Number of 19 *mcr*-positive KpnC isolates; (**b**) *K. pneumoniae*; (**c**) *K. variicola*; and (**d**) *K. quasipneumoniae*. Abbreviations: gentamicin (GEN), amikacin (AMK), amoxicillin/clavulanic acid (AUG), piperacillin/tazobactam (PTZ), cefepime (CPM), cefotaxime (CTX), ciprofloxacin (CIP), levofloxacin (LEV), ertapenem (ETP), imipenem (IMI), meropenem (MEM), ceftazidime (CAZ), chloramphenicol (CHL), tetracycline (TET), fosfomycin (FOT), nitrofurantoin (NIT), azithromycin (ATH), trimethoprim (TMP).

Antimicrobial resistance to 21 antimicrobial agents was characterized in KpnC. Of the 19 *mcr*-harboring KpnC isolates, 13 had intermediate resistance to colistin with MIC values of 1–2 µg/mL (Table 2). Isolates carrying *mcr-8* were more resistant to colistin than *mcr-7*-harboring isolates. As shown in Figure 1, the antimicrobial susceptibility testing of the 19 antimicrobial agents was determined in 19 *mcr*-harboring KpnC isolates. Most of the KpnC isolates were susceptible to levofloxacin, chloramphenicol, ceftazidime, gentamycin, and trimethoprim. *K*. *quasipneumoniae* and *K*. *variicola* were more susceptible to antimicrobials than *K*. *pneumoniae*. All the *mcr*-carrying isolates were susceptible to amoxicillin/clavulanic acid, piperacillin/tazobactam, cefotaxime, cefepime, imipenem, ertapenem, meropenem, amikacin, and fosfomycin. This indicated that *mcr*-harboring KpnC isolated from animals appeared to be highly susceptible to antibiotics including carbapenems (imipenem, ertapenem, meropenem), aminoglycosides (amikacin), fosfomycin, β-lactamase inhibitors (piperacillin/tazobactam, amoxicillin/clavulanic acid), third-generation cephalosporins (cefotaxime), and fourth-generation cephalosporin (cefepime).

### 3.3. Transferability of mcr Genes

To prove the ability of KpnC to transfer *mcr*-harboring plasmid to the different bacterial species, the 19 *mcr*-carrying KpnC isolates were subjected to conjugation assays using *E*. *coli* UB1637 (streptomycin-resistant) as the recipient. Of these, only five KpnC donors exhibited transferability. Among these transconjugants, *mcr-8* (*n* = 4) from *K. pneumoniae* and *mcr-1* (*n* = 1) from *K. quasipneumoniae* were successfully transferred, whereas *mcr-7* was not detected in any of the transconjugants in this study (Table 3). All transconjugants had MIC values for colistin in the range 4–8 µg/mL (Table 3). In addition, donors of *K. pneumoniae* harboring *oqxAB* (*n* = 2) or *bla*_SHV_ (*n* = 2) were co-transferred into recipient *E. coli* cells (Table 3).

### 3.4. MLST Analysis

**Figure 2:**
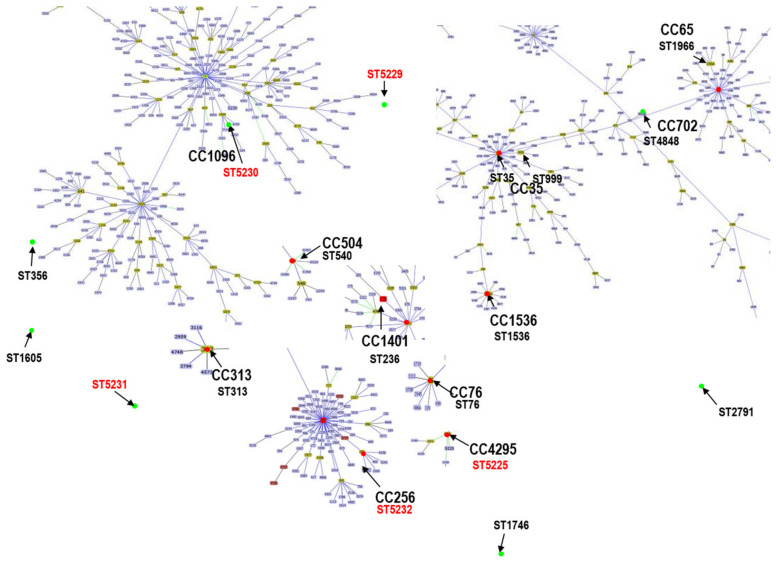
Population snapshot of all KpnC isolates typed in current study using goeBURST analysis compared to entire database of *K. pneumoniae* MLST isolates (8 November 2020). Black letters indicate matching of KpnC STs in this study with database entries. Green dots indicate individual founders. Different clonal complexes are shown. ST5225 and ST5229–ST5232 are novel STs identified in this study and are shown in red.

MLST was carried out on the 19 *mcr*-harboring KpnC isolates. Eighteen STs were assigned to *mcr*-harboring KpnC isolates (Table 4). Five novel STs were defined among these *mcr*-harboring KpnC isolates. These novel STs were identified as ST5225 and ST5229–ST5232 (Table 4). This indicated the diversity of KpnC carrying *mcr* in the current study. The goeBURST analysis identified 11 clonal complexes (CCs) and 6 singletons in our isolates (Figure 2, Table 4). The most prevalent clones were CC35 (ST35 (*n* = 2) and ST999 (*n* = 1)) of *mcr*-harboring KpnC.

## 4. Discussion

Colistin has been extensively used in pig production for the prevention and treatment of diarrhea (especially during the post-weaning period), and as a countermeasure to endemic diseases [38]. Currently, in Thailand, colistin application is prohibited for disease prevention in livestock [39]; however, the dissemination has been reported of a mobile colistin resistance gene such as *mcr* contributing to cross-contamination among carcasses during slaughter and post-slaughter processing [11,21,40]. The current study identified the mobile colistin genes *mcr-8*, *mcr-7*, *mcr-7 + mcr-8*, and *mcr-1* + *mcr-7* in KpnCs, listed in order of prevalence (most to least). Interestingly, this is the first report to demonstrate that *K*. *variicola* and *K*. *quasipneumoniae* from slaughtered pigs harbored *mcr-7* or *mcr-8* genes. Notably, *mcr-1*, *mcr-7*, and *mcr-8* have been identified in *K. pneumoniae* isolates from pigs and chickens [35,41,42,43]. Another Thai study showed that several variants of *mcr* genes (*mcr-1.1*, *-2.3*, *-3.19*, *-3.40*, *-3.5*, and *-8*) were detected in eight *K. pneumoniae* complex isolates from pigs and humans on farms in Thailand [44]. Another reported *E. coli* strains harboring *mcr-1*, *mcr-3*, *mcr-6*, *mcr-7*, *mcr-8*, and *mcr-9*, together with co-occurrences of *mcr-1 + mcr-3*, *mcr-1 + mcr-9*, and *mcr-3 + mcr-6 + mcr-7* isolated from slaughtered pigs in Thailand [23]. These indicated that Enterobacteriaceae harboring *mcr* genes isolated from pigs in Thailand showed a high diversity of *mcr* variants, and this should be taken into account in the development of efficient monitoring systems of AMR bacteria and antimicrobial usage.

We have shown that certain KpnC isolates were able to transfer *mcr-1* or *mcr-8* to an *E. coli* UB1637 recipient, resulting in higher MIC values for colistin than those of the donors. In addition, the *mcr-1* gene was transferred from *K. quasipneumoniae* to *E. coli* recipients with a higher colistin MIC value than for the donor strain. Increased resistance to colistin was observed in *K. pneumoniae* of swine origin and the recipient *E. coli* DH5α in terms of the *mcr-1* gene [36]. These findings suggest that *E. coli* UB1637 is a host strain that is likely to support enhanced *mcr* expression compared to KpnC. Co-transfer of *mcr-8* + *bla*_SHV_ + *oqxAB* was found in two isolates. This suggested that, in this study, the spread of *mcr* or β-lactamase or PMQR genes could be transmitted to other pathogens and could spread to other hosts.

KpnCs (especially involving *K. pneumoniae*) from slaughtered pigs that harbored *mcr* genes were highly resistant to azithromycin. Macrolides are important antimicrobials for the treatment of infections in cattle and pigs and are commonly used in pig farms worldwide [45]. Resistance to macrolide of *K. pneumoniae* from pigs has also been reported in Thailand, with the most resistant gene being *mdf(A)* [44]. However, all our isolates were susceptible to carbapenem, amoxicillin/clavulanic acid piperacillin/tazobactam, and third-generation cephalosporins, which was consistent with other studies [46,47].

The current study showed that *mcr* isolates exhibited high diversity, as revealed by MLST analysis, and the STs of 26.3% (5/19) KpnC isolates were novel. The goeBURST analysis showed that ST999 had a single-locus variant of ST35 [30]. This ST has been identified in OXA-48-producing *K. pneumoniae* isolated from humans [48], whereas our isolate carried *mcr-8*. *K. pneumoniae* ST35, a global multidrug-resistant clone that has been isolated in many countries [49,50,51]. In Thailand, ST35 harboring *mcr-3* have been found in humans [44]. The current study detected ST35 in slaughtered pigs, emphasizing that pigs and human *K. pneumoniae* isolates might be from the same source, and pig-borne transmission plays a crucial role in the transmission of *mcr*-carrying *K. pneumoniae*.

## 5. Conclusions

The study revealed colistin non-susceptible KpnC harboring *mcr-1, mcr-7*, and *mcr-8* genes in slaughtered pigs in Thailand. Therefore, improvements are strongly recommended in food hygiene standards and biosecurity measures on farms and in slaughtering procedures. This evidence-based knowledge of *mcr*-harboring KpnC in slaughtered pigs can be applied in livestock policy planning, monitoring changes in population dynamics, and the development of surveillance strategies for prevention and control programs.

## Figures and Tables

**Table 1:** Profiles of antimicrobial resistance genes found in KpnC isolates from slaughtered pigs in Thailand.

Species	Mobile Colistin Resistance Genes (*mcr*) (%) ^a^				β-lactamases Genes (%) ^b^							PMQR (%) ^c^		
	*mcr-7*	*mcr-8*	*mcr-7 + mcr-8*	*mcr-1 + mcr-7*	*bla*_TEM_	*bla*_SHV_	*bla*_CTX-M_	*bla*_TEM_ + *bla*_SHV_	*bla*_TEM_ + *bla*_CTX-M_	*bla*_CTX-M_ + *bla*_SHV_	*bla*_TEM_ + *bla*_SHV_ + *bla*_CTX-M_	*Oqx AB*	*qnrS*	*Oqx AB +* *qnr S*
*K. pneumoniae*	6 (2.14)	8 (2.85)	2 (0.71)	-	4 (1.43)	184 (65.71)	1 (0.36)	33 (11.79)	-	3 (1.07)	3 (1.07)	168 (60.00)	4 (1.43)	36 (12.86)
*K. variicola*	1 (0.36)	-	-	-	7 (2.50)	-	5 (1.78)	-	1 (0.36)	-	-	7 (2.50)	-	2 (0.71)
*K. quasipneumoniae*	-	1 (0.36)	-	1 (0.36)	4 (1.43)	3 (1.07)	-	-	-	-	-	3 (1.07)	-	-
Total	7 (2.50)	9 (3.21)	2 (0.71)	1 (0.36)	15 (5.36)	187 (66.79)	6 (2.14)	33 (11.79)	1(0.36)	3 (1.07)	3 (1.07)	178 (63.57)	4 (1.43)	38 (13.57)

^a^ Profile of antimicrobial resistance genes (*mcr*) in KpnC isolates. ^b^ Profile of antimicrobial resistance genes (β-lactamases) in KpnC isolates. ^c^ Profile of antimicrobial resistance genes (PMQR) in KpnC isolates. Abbreviations: +, co-existence of antimicrobial-resistance genes.

**Table 2:** MIC values of colistin in KpnC harboring *mcr* genes.

Mobile Colistin Resistance Genes ^a^	Species	N (%)	No. of Isolates at MIC of Colistin (%)	
			≤2 µg/mL (I)	≥4 µg/mL (R)
*mcr-* *8*	*K. pneumoniae*	8 (42.10)	3 (15.78)	5 (26.31)
	*K. variicola*	-	-	-
	*K. quasipneumoniae*	1 (5.26)	1 (5.26)	-
*mcr-7*	*K. pneumoniae*	6 (31.57)	5 (26.31)	1 (5.26)
	*K. variicola*	1 (5.26)	1 (5.26)	-
*mcr-7 + mcr-8*	*K. pneumoniae*	2 (10.52)	2 (10.52)	-
*mcr-1 + mcr-7*	*K. quasipneumoniae*	1 (5.26)	1 (5.26)	-
Total		19 (100)	13 (68.42)	6 (31.57)

^a^ Profile of mobile colistin resistance genes in KpnC isolates. Abbreviations: I, intermediate; R, resistant; +, co-existence of antimicrobial-resistance genes; -, not found.

**Table 3:** Profiles of antimicrobial-resistance genes in KpnC of donors *E. coli* and tranconjugants.

Donor Species	Pattern of *mcr D*onor ^a^	MIC ^b^		Gene Transfer ^c^		
		Donor	Trans-conjugant	*mcr*	β-lactamases	PMQR
*K. pneumoniae*	*bla*_SHV_ + *OqxAB + mcr-8*	2	8	*mcr-8*	*bla*_SHV_	*oqxAB*
		2	8	*mcr-8*	-	-
		4	8	*mcr-8*	-	-
	*bla*_TEM_ + *bla*_SHV_ + *OqxAB + qnrS + mcr-8*	4	4	*mcr-8*	*bla*_SHV_	*oqxAB*
*K. variicola*	*bla*_TEM_ + *bla*_SHV_ *+ mcr-7*	-	-	*-*	-	*-*
*K. quasipneumoniae*	*bla*_TEM_ *+ mcr-* *1 +* *mcr-7*	2	4	*mcr-1*	-	-
Total			5 (26.32)	5 (26.32)	2 (10.53)	2 (10.53)

^a^ Pattern of antimicrobial resistance genes in KpnC donor strains. ^b^ MIC values of colistin in KpnC donor and tranconjugant strains. ^c^ Profile of antimicrobial-resistance genes (*mcr*, β-lactamases, and PMQR) in tranconjugants. Abbreviations: +, co-existence of antimicrobial-resistance genes; -, not found.

**Table 4:** Antimicrobial resistance profiles of KpnC isolated from slaughtered pigs in Thailand.

Species	ID	Pattern of Resistance Genes ^a^	Total	% (n = 19)	Pattern of Resistance Antimicrobial ^b^	Total	ST ^c^	CC
*K. pneumoniae*	57 RB	*bla*_SHV_ + *oqx AB* + *mcr-8*	4	21.05	ATH-CO	1 (5.26)	76	CC76
	15 K.SK				ATH-TET-CO	1 (5.26)	5229	Singleton
	9 K.SK				ATH-NIT	1 (5.26)	1746	Singleton
	7 K.SK				-	1 (5.26)	35	CC35
	20 K.SK	*oqx AB* + *mcr-8*	1	5.26	ATH-CO	1 (5.26)	999	CC35
	40 K.SK	*bla*_SHV_ + *oqx AB* + *mcr-7*	4	21.05	ATH	2 (10.53)	540	CC504
	7 K.PKK						5225	CC4295
	17 K.CM				ATH-CO	1 (5.26)	35	CC35
	46 K.CM				TET	1 (5.26)	1966	CC65
	64 BK	*bla*_SHV_ + *bla*_TEM_ + *oqx AB* +*mcr-8*	1	5.26	ATH-NIT-CO	1 (5.26)	2791	Singleton
	43 K.SK	*bla*_SHV_ + *oqx AB* + *MCR-8* + *mcr-7*	2	10.53	ATH-TET	1 (5.26)	313	CC313
	30 K.CM				CHI-TET-TMP	1 (5.26)	236	CC1401
	69 BK	*bla*_SHV_ + *bla*_TEM_ + *oqx AB* + *qnrS* + *mcr-8*	2	10.53	CAZ-GEN-ATH-CIP-CHI	1 (5.26)	5232	CC256
	13 K.CM				GEN-TET-ATH-CIP-LEV-CO	1 (5.26)	5231	Singleton
	7 NP	*bla*_SHV_ + *oqx AB* + *qnrS* + *mcr-7*	2	10.53	NIT-CIP-LEV	1 (5.26)	1536	CC1536
	30 NP				ATH-NIT	1 (5.26)	4848	CC702
*K. variicola*	34 K.KK	*bla*_TEM_ + *oqx AB* + *mcr-7*	1	5.26	ATH-NIT	1 (5.26)	5230	CC1096
*K. quasipneumoniae*	11K.KK	*bla*_TEM_ *+ mcr-8*	1	5.26	ATH	1 (5.26)	1605	Singleton
	24K.SK	*bla*_TEM_ *+ mcr-1 + mcr-7*	1	5.26	ATH-NIT	1 (5.26)	356	Singleton

^a^ Pattern of antimicrobial-resistance genes in KpnC donor strains. ^b^ Abbreviations: gentamicin (GEN), amikacin (AMK), amoxicillin/clavulanic acid (AUG), piperacillin/tazobactam (PTZ), cefepime (CPM), cefotaxime (CTX), ciprofloxacin (CIP), levofloxacin (LEV), ertapenem (ETP), imipenem (IMI), meropenem (MEM), ceftazidime (CAZ), chloramphenicol (CHL), tetracycline (TET), fosfomycin (FOT), nitrofurantoin (NIT), azithromycin (ATH), trimethoprim (TMP), CC, clonal complex. ^c^ MLST was performed on 19 *mcr*-positive KpnC isolates.

## Data Availability

Not applicable.
